# Construction and Biological Characteristics of a Quadruple Gene-Deleted Strain of Orf Virus as a Vaccine Candidate

**DOI:** 10.3390/v17060760

**Published:** 2025-05-27

**Authors:** Jiawen Zhang, Ruolan Xin, Junjie Zhao, Ruizhi Wu, Daoming Su, Menglin Li, Yuanyuan Zhu, Xiaoyun Chen, Zhen Zhu

**Affiliations:** China Institute of Veterinary Drug Control, Beijing 100086, China; 15170542592@163.com (J.Z.); ttkxxrl@163.com (R.X.); zhaojunjie_ivdc@163.com (J.Z.); 15930269929@163.com (R.W.); sudaoming123@outlook.com (D.S.); 19853505816@163.com (M.L.); zhuyuanyzz@163.com (Y.Z.); caucxy@163.com (X.C.)

**Keywords:** ORFV, VIL-10 gene, rGS14-QuadMut, safety, vaccine candidate

## Abstract

(1) Background: Contagious ecthyma, also known as orf, is an epitheliotropic zoonotic disease caused by the orf virus (ORFV), primarily affecting the skin and mucous membranes of ruminants such as goats and sheep, leading to the formation of papules and pustules. Vaccination is the most effective way to prevent this disease in susceptible animals; however, traditional attenuated vaccines carry the potential risk of reversion to virulence. Therefore, there is an urgent need to develop safe and effective vaccines for the prevention and control of orf. (2) Methods: In this study, building upon the previously constructed ORFV three-gene deletion strain rGS14-TrypMut, we employed homologous recombination to knock out the VIL-10 gene and successfully constructed a four-gene deletion strain, rGS14-QuadMut. We evaluated its in vitro growth characteristics, safety, and protective efficacy in a challenge model. (3) Results: The in vitro results show that rGS14-QuadMut had a replication ability similar to that of other two-gene deletion strains, with good genetic stability. In in vivo experiments, compared to rGS14-TrypMut, rGS14-QuadMut caused only mild redness and swelling at the inoculation site, with a faster healing rate, indicating better safety. Additionally, rGS14-QuadMut induced strong differentiation of CD4^+^ and CD8^+^ T cells, increased the CD4^+^/CD8^+^ ratio, and primarily stimulated a Th1-type immune response, with significant changes in cytokine levels, including IL-8, IFN-γ, and IL-2. In the challenge protection experiment, both rGS14-QuadMut and rGS14-TrypMut provided 100% protective efficacy. In conclusion, rGS14-QuadMut demonstrated enhanced safety without compromising immune protection efficacy and is a promising candidate for an orf live vaccine strain.

## 1. Introduction

Orf, also known as contagious ecthyma (CE), is a zoonotic disease caused by the orf virus (ORFV) [1]. ORFV belongs to the Poxviridae family and the Parapoxvirus genus. It is a double-stranded DNA virus with a genome size of approximately 138 kb. The virus particles are mainly oval, with surface capsid proteins arranged in a tubular or filamentous pattern, and an outer lipid membrane [2,3]. ORFV primarily infects small ruminants such as goats and sheep, though it can also affect other herbivorous animals. The typical clinical manifestations include the formation of papules, vesicles, pustules, and warty scabs on the skin and mucous membranes around the lips, oral mucosa, nostrils, and udder [4]. ORFV not only reduces milk production in affected ruminants, but also severely impacts their ability to feed, leading to weight loss, weakness, and, in severe cases, death [5]. The disease is characterized by high morbidity but low mortality, though repeated infections are common, which is attributed to the virus’s immune evasion mechanisms [6,7]. Furthermore, animals infected with orf are more susceptible to secondary bacterial infections, particularly Staphylococcus aureus, which increases the mortality rate in infected animals and causes significant economic losses in the sheep farming industry [8,9].

Vaccination is currently the most effective strategy for the prevention and control of ORF, as it can significantly reduce the incidence of disease and the risk of viral transmission [10]. Since 1981, live attenuated vaccines have been the primary option for preventing ORF [11]. The only commercial ORF vaccine in China is weakened by cell passaging, but it does retain some residual virulence [12]. Compared with traditional live vaccines with reduced virulence, gene-deletion vaccines knock out virulence genes through genetic engineering technology, thereby reducing the virulence of the virus, and have higher safety and controllability. At the same time, they retain immune protective efficacy, making them the focus of current research on ORF virus vaccines.

During the viral infection phase, the virus expresses various immune-modulatory proteins, such as interleukin-10 homolog (ORFV127), chemokine binding protein (CBP), granulocyte–macrophage colony-stimulating factor inhibitory protein (GIF), immunomodulators of the nuclear factor kappa (NF-κB) signaling pathway (e.g., ORFV121), and vascular endothelial growth factor (VEGF). These proteins play significant roles in regulating host tropism, viral virulence, immune modulation, pathogenicity, and the maturation of viral assembly [4,13]. GIF inhibits the functions of IL-2 and GM-CSF, thereby preventing the activation of leukocytes and dendritic cells (DCs); CBP can suppress the migration of immature and mature DCs in response to inflammatory and constitutive chemokines; ORFV121 reduces the phosphorylation of NF-κB-P65, inhibiting the NF-κB signaling pathway and thus suppressing the inflammatory response; and ORFV127 encodes the VIL-10 protein, a key immune escape factor that inhibits the migration of monocytes and mast cells to sites of skin inflammation, suppresses the synthesis of inflammatory cytokines, inhibits antigen presentation, and indirectly suppresses T-cell activation, including IL-1β, IL-8, and TNF-α. Furthermore, VIL-10 also has the ability to suppress IFN-γ expression in the natural host, thereby exacerbating skin damage in infected sheep [14,15,16,17,18,19].

In this study, based on the previously successfully constructed ovine parapoxvirus three-gene knockout strain rGS14-TrypMut, we aimed to further knockout the VIL-10 gene to construct a four-gene knockout strain, rGS14-QuadMut. By assessing its in vitro growth characteristics, safety, immunogenicity, and immune protection rate, we evaluate its potential as a live vaccine candidate for CE. The results indicate that rGS14-QuadMut demonstrated enhanced safety, promoted a higher level of CD4^+^ differentiation, and increased the CD4^+^/CD8^+^ ratio. Significant changes in the levels of IL-8, IFN-γ, and IL-2 cytokines were observed, and the strain primarily induced a Th1-type immune response. These findings suggest that rGS14-QuadMut has the potential to become a viable live vaccine candidate for CE in sheep.

## 2. Materials and Methods

### 2.1. Virus and Cells

The ORFV-GS14 strain (isolated from a goat with CE in Gansu Province, China, and named GS14) is stored at the China Veterinary Drug Control Institute and was used as the parental virus throughout the experiment. The orf virus CQ strain (ORFV-CQ), isolated and identified from clinical samples of goats in the Chongqing region of China, is preserved at the China Institute of Veterinary Drug Control. This strain underwent five serial passages in PLT cells and was subsequently stored in our laboratory. ORFV-CQ was used as the challenge strain in the challenge protection experiment. The viral stock used in the experiment was the product obtained after five passages in PLT cells and was stored at −80 °C for future use. The ORFV three-gene knockout strain rGS14-TrypMut was previously constructed and stored in our laboratory [20,21]. Primary lamb testicular (PLT) cells were prepared and preserved in our laboratory. PLT cells were cultured in Ham’s DMEM/12 Gluta MAX medium (Thermo Fisher Scientific, New York, NY, USA) containing 10% fetal bovine serum (Biosharp, Hefei, Anhui, China) at 37 °C with 5% CO_2_.

### 2.2. Construction of rGS14-QuadMut

#### 2.2.1. Primer Design

Primers were designed based on the ORFV rGS14 genomic sequence and synthesized by Sangon Biotech (Shanghai, China) Co., Ltd. (Table 1).

#### 2.2.2. Construction of the Homologous Recombination Fragment

**Figure 1:**
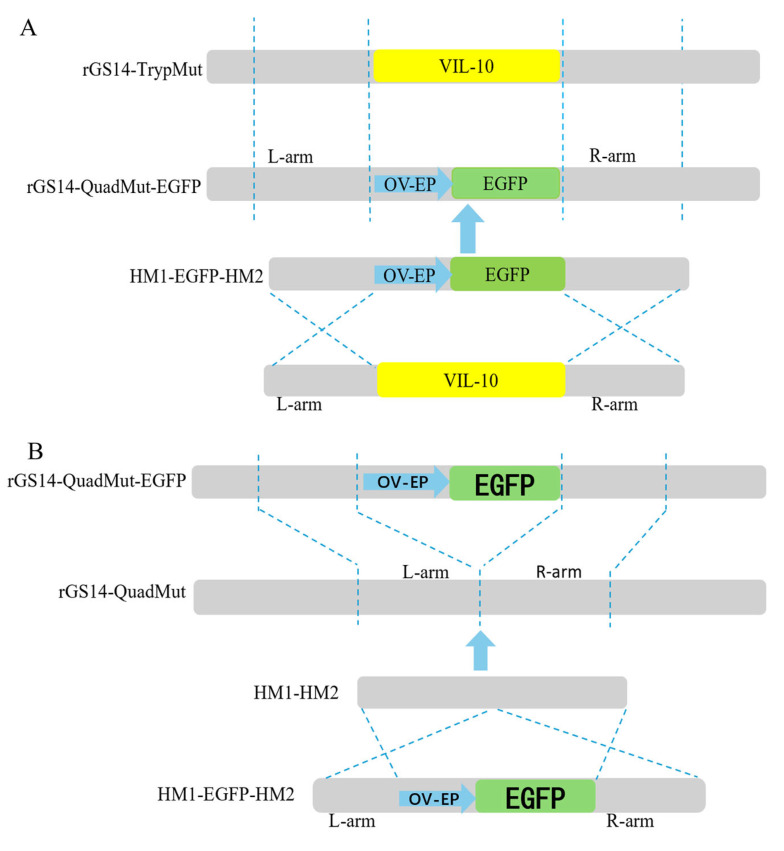
(**A**) Schematic diagram for the construction of rGS14-QuadMut-OV-EP-EGFP; (**B**) schematic diagram for the construction of rGS14-QuadMut.

The construction strategy is shown in Figure 1A. First, PCR amplifications were carried out, respectively, using three pairs of primers, namely VIL-10-hm1-F/VIL-10-hm1-egfp-R, VIL-10-hm2-egfp-F/VIL-10-hm2-R, and egfp-VIL-10-hm1-F/egfp-VIL-10-hm2-R, to obtain the left and right homologous arms containing the overlapping fragments and the reporter gene (OV-EP/EGFP) flanked by the left and right arms of the viral interleukin-10 (vIL-10). Then, using VIL-10-hm1-F/VIL-10-hm2-R as primers, the fusion fragment consisting of the left and right homologous arms of vIL-10 and OV-EP/EGFP was obtained by fusion PCR technology, and it was named HM1-EGFP-HM2. This fusion fragment was sent to Shanghai Sangon Biotechnology Co., Ltd. for sequencing and alignment. The results confirm the successful construction of the HM1-EGFP-HM2 fragment. The fragment was stored at −20 °C for subsequent use.

The construction strategy is shown in Figure 1B. By constructing the homologous recombination fragment HM1-HM2, the OV-EP/EGFP reporter gene was knocked out based on the principle of homologous recombination. Two pairs of primers, VIL-10-hm1-hm2-F/VIL-10-hm1-hm2-R and VIL-10-hm2-hm1-F/VIL-10-hm2-hm1-R, were used for PCR amplification, respectively, to obtain the left and right homologous arms containing overlapping fragments. Then, using VIL-10-hm1-hm2-F/VIL-10-hm2-hm1-R as primers and the left and right homologous arms containing overlapping fragments as templates, the fusion fragment HM1-HM2, composed of the left and right homologous arms of vIL-10, was obtained through fusion PCR technology. The fragment was sent for sequencing and alignment at Shanghai sangon Biotechnology Co., Ltd., and successful construction of the HM1-HM2 fragment was confirmed. The fragment was stored at −20 °C for later use.

#### 2.2.3. Construction of rGS14-QuadMut-EGFP

The three-gene knockout strain was infected into PLT cells at an MOI of 0.1. After 2 h of infection, 4 μg of the homologous recombination fusion fragment HM1-EGFP-HM2 was transfected into the infected cells using 10 μL of LipofectamineTM 3000 reagent (Thermo Fisher Scientific, New York, NY, USA) and 5 μL of P3000TM reagent (Thermo Fisher Scientific, New York, NY, USA). The cells were incubated at 37 °C for 8 h, and the growth medium (Ham’s DMEM/12 Gluta MAX medium supplemented with 2% FBS) was replenished. After 48 h, cells exhibiting green fluorescence were observed under a fluorescence microscope, and the areas of cytopathic effect (CPE) with fluorescence were selected. Through three rounds of limiting dilution, until all CPE s exhibited green fluorescence, cells were repeatedly frozen and thawed three times. Viral DNA was then extracted, and PCR amplification was performed to verify the deletion of the VIL-10 gene.

#### 2.2.4. Construction of rGS14-QuadMut

To remove the reporter sequences from rGS14-QuadMut-EGFP, the HM1-HM2 fusion fragment was transfected into PLT cells infected with rGS14-QuadMut-EGFP according to the instructions of the transfection reagent kit. After transfection, the cells were observed under a fluorescence microscope, and those showing no green fluorescence at the lesion sites were selected. The cytopathic clumps that do not emit green fluorescence were picked out and pipetted into the EP tube filled with liquid repeatedly to ensure that the cell clumps were fully integrated into the liquid, and the EP tube was repeatedly frozen and thawed 3 times. The viral solution was diluted from 10^−1^ to 10^−7^ and seeded into 96-well plates pre-plated with PLT cells, and the lesions of green fluorescent cells in each well were observed daily by fluorescence microscopy. Repeat the above 5 times until the lesion no longer has green fluorescence under microscopic observation. The absence of EGFP sequences in rGS14-QuadMut was verified by PCR.

#### 2.2.5. Construction of rGS14∆VIL-10

Consistent with the method described in Section 2.2.3, the EGFP gene was used to replace the VIL-10 gene of the rGS14 strain by using the principle of homologous recombination to construct rGS14∆VIL-10-EGFP, and the purified rGS14∆VIL-10-EGFP was successfully obtained by 5 rounds of limiting dilution, and the VIL-10 gene was not detected in rGS14∆VIL-10-EGFP, but the EGFP gene was detected.

The aforementioned HM1-HM2 homologous recombination fragment was then transfected into PLT cells infected with rGS14∆VIL-10-EGFP to knock out the EGFP gene to obtain rGS14∆VIL-10. Purification of recombinant viruses rGS14∆VIL-10 required 4 rounds of limiting dilution purification, and no EGFP ∆ VIL-10 genes were detected in either purified rGS14 VIL-10.

### 2.3. Determination of in Vitro Growth Characteristics of rGS14-QuadMut

To determine half of the tissue culture infective dose (TCID_50_) of rGS14-QuadMut, rGS14-TrypMut, and rGS14∆VIL-10, each of the three recombinant viruses were seeded into a 96-well plate covered with a monolayer of PLT cells (8 wells of 100 μL per well at each dilution).After incubating at 37 °C with 5% CO_2_ for 2 h, the maintenance medium was added, and cells were cultured continuously. From day 4 onward, the number of wells exhibiting CPE was recorded, and the TCID_50_ was calculated using the Reed–Muench method.

To evaluate the replication capacity of different gene knockout strains, rGS14-QuadMut, rGS14-TrypMut, and rGS14∆VIL-10 were inoculated into T25 flasks containing confluent monolayers of PLT cells at an MOI of 1.0. After three repeated freeze–thaw cycles at 6 h, 12 h, 24 h, 48 h, 72 h, 96 h, 144 h, and 192 h after infection, the repeated freeze–thaw liquid was filtered through a 0.22 μm filter to collect the viral solution, and the TCID_50_ was calculated to determine the viral titer and then the multi-step growth curve was plotted. Viral DNA from viral fluids collected at various time points was extracted using a viral DNA extraction kit and quantified by a quantitative polymerase chain reaction (qPCR) established in the previous laboratory [22]. A viral nucleic acid copy number change curve was plotted to further analyze the replication kinetics of different gene knockout strains. To examine the cytopathic differences of rGS14-QuadMut, rGS14-TrypMut, and rGS14∆VIL-10 in PLT cells, each virus was diluted to the same TCID50, and then an equal amount of the diluted viruses was inoculated into a 6-well plate of PLT cells that are in good growth condition and have a uniform cell density. Subsequently, the plate was placed in a cell incubator for cultivation. The morphological changes in the cells were observed and recorded under an inverted microscope at 24, 48, and 72 h post-infection. This allowed for the analysis of CPE differences among viruses among the different gene knockout strains by comparing the cytopathic effects.

To verify the genetic stability of rGS14-QuadMut, it was passaged continuously in PLT cells. The purified four-gene knockout strain was designated as the first generation. Virus samples from the 5th, 10th, 15th, 20th, and 25th generations were randomly selected. After three freeze–thaw cycles, viral genomic DNA was extracted, and PCR amplification, sequencing, and alignment were performed using two pairs of primers (VIL-10 F/R and VIL-10-hm1-F/VIL-10-hm1-R) to verify the genetic stability of the virus.

### 2.4. Safety Experiment

Nine one-month-old lambs, negative for both ORFV antigen and antibody, were randomly divided into three groups: the rGS14-QuadMut immunization group, the rGS14-TrypMut immunization group, and the blank control group, with three animals per group. The two experimental groups were inoculated with the corresponding virus suspensions via the intradermal scratch method on the inner thigh, with a dosage of 0.2 mL containing 10^6^ TCID_50_ per lamb. The control group was inoculated with the same volume of DMEM/F12 medium. The inoculation site on the inner thigh was photographed daily, and clinical symptoms at the inoculation site were scored. Clinical scoring was based on the method of Martins [23], using four clinical indicators: erythema and swelling, vesiculation and/or pustules, hemorrhage and/or exudation, and scab formation. These symptoms were used as criteria for assessment, and scores were quantified according to the severity of the symptoms.

### 2.5. T-Cell Subset Detection

Blood samples were collected from the lambs before immunization (day 0) and on day 21 after immunization. Peripheral blood mononuclear cells (PBMCs) were isolated using a goat peripheral blood mononuclear cell isolation kit according to the manufacturer’s instructions. The isolated cells were washed repeatedly with PBS, and after centrifugation, the supernatant was discarded, and the cells were resuspended to determine the cell concentration. The cell concentration was adjusted to 1 × 10^6^/100 μL, and 10 μL of the corresponding flow cytometry antibody was added to each sample tube. The cells were incubated on ice in the dark for 30 min. Following incubation, the cells were washed by adding 150 μL of PBS and then centrifuged at 400× *g* for 5 min at room temperature. After discarding the supernatant, the washing procedure was performed again. Finally, the cells were resuspended in 300 μL PBS and stored on ice while being protected from light. Before conducting flow cytometry, the cell suspension was passed through a 70 μm filter and placed into flow cytometry tubes for subsequent analysis. The BD flow cytometer was turned on and the established calibration protocol was followed. First, the instrument was calibrated with standard beads, and then the cells in the blank control group were detected, and the relevant parameters of the instrument were further adjusted to make the fluorescence signal intensity of the negative cell population stable at about 10^−^², thus laying the foundation for subsequent accurate detection. After the instrument parameters were adjusted, the cells in the experimental group were detected. During the assay, the fluorescence signal of the cells was acquired using FACSDiva software v9.0 (BD Biosciences, New York, USA), and after the data acquisition was complete, it was imported into the FlowJo software v10 (BD Biosciences, New York, USA) for data analysis. First, all lymphocytes were circled by FSC-A and SSC-A, and then the cells were de-adhered by FSC-A and FSC-H, and then the proportion of target cells was determined according to the corresponding antibodies.

### 2.6. Cytokine Detection

Serum samples were obtained from the jugular vein of the lambs at various time points post-immunization (0, 3, 5, 7, 10, 14, and 21 days). Serum cytokine levels were measured using a cytokine ELISA kit (sheep Interleukin 10 (IL-10) ELISA Kit, sheep Interleukin-2 (IL-2) ELISA Kit, sheep In-terleukin 8 (IL-8) ELISA Kit, sheep Interferon γ (IFN-γ) ELISA Kit, all are sourced from Cusabio Biotech Co., Ltd in Wuhan, China). Data were recorded to evaluate the dynamic variations in the immune response of the lambs over the specified time periods.

### 2.7. Challenge Protection Experiment

To assess the immune protection efficacy, a challenge experiment was performed after immunization. The ORFV-CQ strain was inoculated into the hairless area on the inner thigh of the lambs via the scratch method, with a dosage of 0.2 mL 10^7.0^ TCID_50_/mL. In the early stage of the experiment, the challenge model was constructed for this strain, and the experiment proved that the minimum pathogenic dose of this strain was 0.2mL 10^7.0^ TCID_50_/mL. Based on the above experimental results, the challenge protection experiment was carried out according to the minimum challenge amount, the clinical lesions at the inoculation site were monitored for 14 days, and photos were taken to record them, the scab and surrounding tissues of the inoculation’s scratch on the inner thigh were taken and ground into a tissue suspension, and the sample DNA was extracted after repeated freezing and thawing 3 times, and the viral load of the challenged site was detected by qPCR.

### 2.8. Data Analysis

All experimental data were evaluated using two-way analysis of variance (ANOVA) in GraphPad 6.0 software with statistical significance determined as * *p* < 0.05, ** *p* < 0.01, *** *p* < 0.001, and **** *p* < 0.0001.

## 3. Results

### 3.1. Construction of rGS14-QuadMut and rGS14∆VIL-10

To study the effects of the deletion of the VIL-10 gene on virus replication, virulence, and protective effects, the rGS14-QuadMut-EGFP strain was constructed based on rGS14-TrypMut using homologous recombination, as shown in the construction strategy (Figure 1A). The recombinant virus was purified by three rounds of limiting dilution(see Supplementary Figure S1 for partial results graphs), and rGS14-QuadMut-EGFP was successfully obtained, and its genomic DNA was extracted, followed by polymerase chain reaction (PCR) analysis using two pairs of primers, VIL-10F/YL-10F-10-HM1-R and egfp-VIL-10-HM2-R. Sequencing results confirm that the VIL-10 gene was successfully replaced by the EGFP gene, indicating the successful construction of rGS14-QuadMut-EGFP(see Supplementary Figure S2 for partial results graphs). The EGFP gene was knocked out using the HM1-HM2 fusion fragment by homologous recombination to generate the recombinant virus rGS14-QuadMut, as shown in Figure 1B. The virus was then purified by five rounds of limiting dilution(see Supplementary Figure S3 for partial results graphs)., and the successfully purified rGS14-QuadMut PCR amplification electrophoresis sequencing detected deletions of both the VIL-10 gene and the EGFP gene, indicating that the rGS14-QuadMut virus was successfully constructed and purified (see Supplementary Figure S4 for partial results graphs).

The EGFP gene was replaced by the VIL-10 gene of the GS14 strain by using the principle of homologous recombination to construct rGS14∆VIL-10-EGFP, and the purified rGS14∆VIL-10-EGFP was successfully obtained by five rounds of limiting dilution purification, and the VIL-10 gene was not detected in rGS14∆VIL-10-EGFP, but the EGFP gene was detected. The aforementioned HM1-HM2 homologous recombination fragment was then transfected into PLT cells infected with rGS14∆VIL-10-EGFP to knock out the EGFP gene to obtain rGS14∆VIL-10. Purification of recombinant viruses rGS14∆VIL-10 required four rounds of limiting dilution purification, and no EGFP∆VIL-10 genes were detected in either purified rGS14∆VIL-10.

### 3.2. In Vitro Growth Characteristics of rGS14-QuadMut

**Figure 2:**
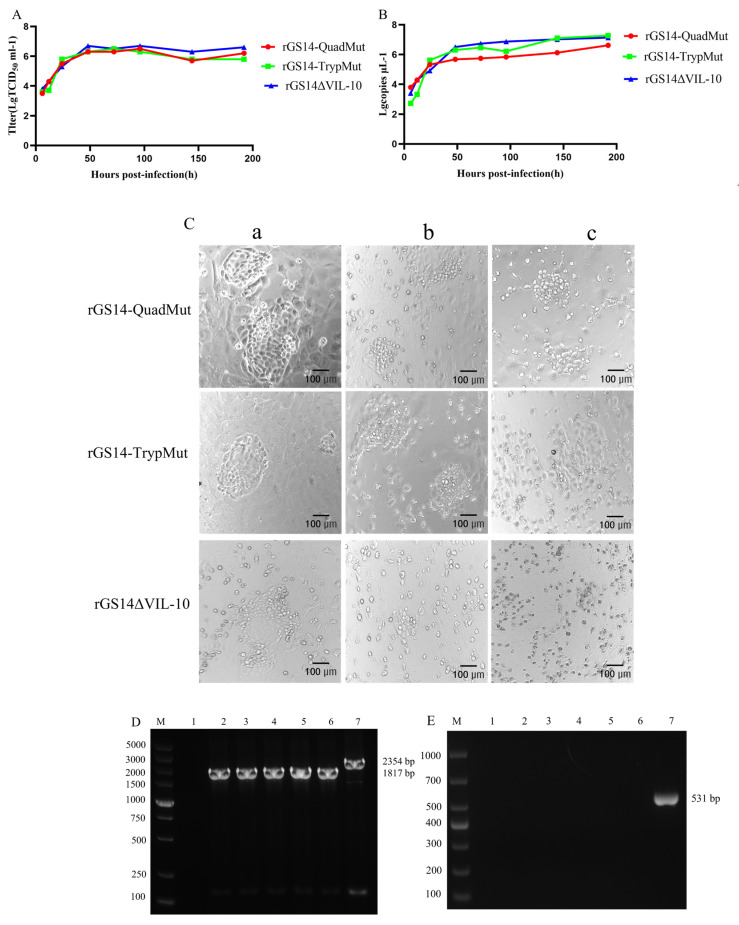
Comparison of in vitro growth characteristics of rGS14-QuadMut, rGS14-TrypMut, and rGS14∆VIL-10. (**A**) Multiple-step growth curves of rGS14-QuadMut, rGS14-TrypMut, and rGS14∆VIL-10; (**B**) viral content curves for rGS14-QuadMut, rGS14-TrypMut, and rGS14∆VIL-10; (**C**) cytopathic effects (CPE) of rGS14-QuadMut, rGS14-TrypMut, and rGS14∆VIL-10 at different time points: (a) 24 h; (b) 48 h; and (c) 72 h; and (**D**) PCR electrophoresis of rGS14-QuadMut using VIL-10-hm1-F/VIL-10-hm2-R VIL-10-hm1-F/VIL-10-hm2-R primers. MV: DL5000 DNA marker; 1: negative control; 2: 5th generation; 3: 10th generation; 4: 15th generation; 5: 20th generation; 6: 25th generation; 7: positive control; and (**E**) PCR validation of VIL-10 gene knockout in rGS14-QuadMut. MV: DL1000 DNA marker; 1: negative control; 2: 5th generation; 3: 10th generation; 4: 15th generation; 5: 20th generation; 6: 25th generation; and 7: positive control.

To compare the replication dynamics of the recombinant viruses rGS14-QuadMut, rGS14-TrypMut, and rGS14∆VIL-10, the TCID_50_ of rGS14-QuadMut, rGS14-TrypMut, and rGS14∆VIL-10 was 10^6.5^ TCID_50_/mL, 10^6.6^ TCID_50_/mL, and 10^6.3^ TCID_50_/mL, respectively. The results of infecting PLT cells with each of the three recombinant viruses with a multiplicity of infection (MOI) of 1.0 (Figure 2A) show that there were no significant differences in the in vitro replication kinetics of the three recombinant viruses. Viral genomic DNA was extracted from samples collected at different time points, and the viral content was quantified by qPCR. The results (Figure 2B) demonstrate that the viral growth trends were similar across all three viruses, with no significant differences. To compare the cytopathic effects at the same time point and infection dose, cells infected with rGS14-QuadMut, rGS14-TrypMut, and rGS14∆VIL-10 were observed for morphological changes. The results (Figure 2C) show that rGS14-QuadMut and rGS14-TrypMut produced similar cytopathic effects at different time points, while rGS14∆VIL-10 exhibited more cell debris compared to both rGS14-QuadMut and rGS14-TrypMut. Additionally, to assess the genetic stability of rGS14-QuadMut across different generations, PCR amplification was performed using the VIL-10F/R and VIL-10-hm1-F/VIL-10-hm2-R primer pairs. The results (Figure 2D) indicate the successful construction and genetic stability of the recombinant virus. This suggests that the recombinant virus holds potential for further experimentation and application as a candidate vaccine.

### 3.3. Safety Evaluation of rGS14-QuadMut

**Figure 3:**
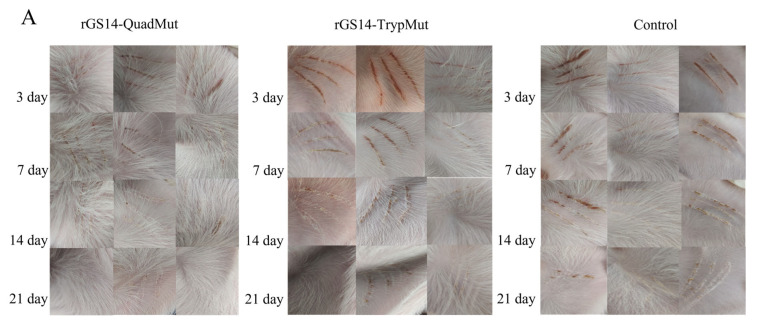

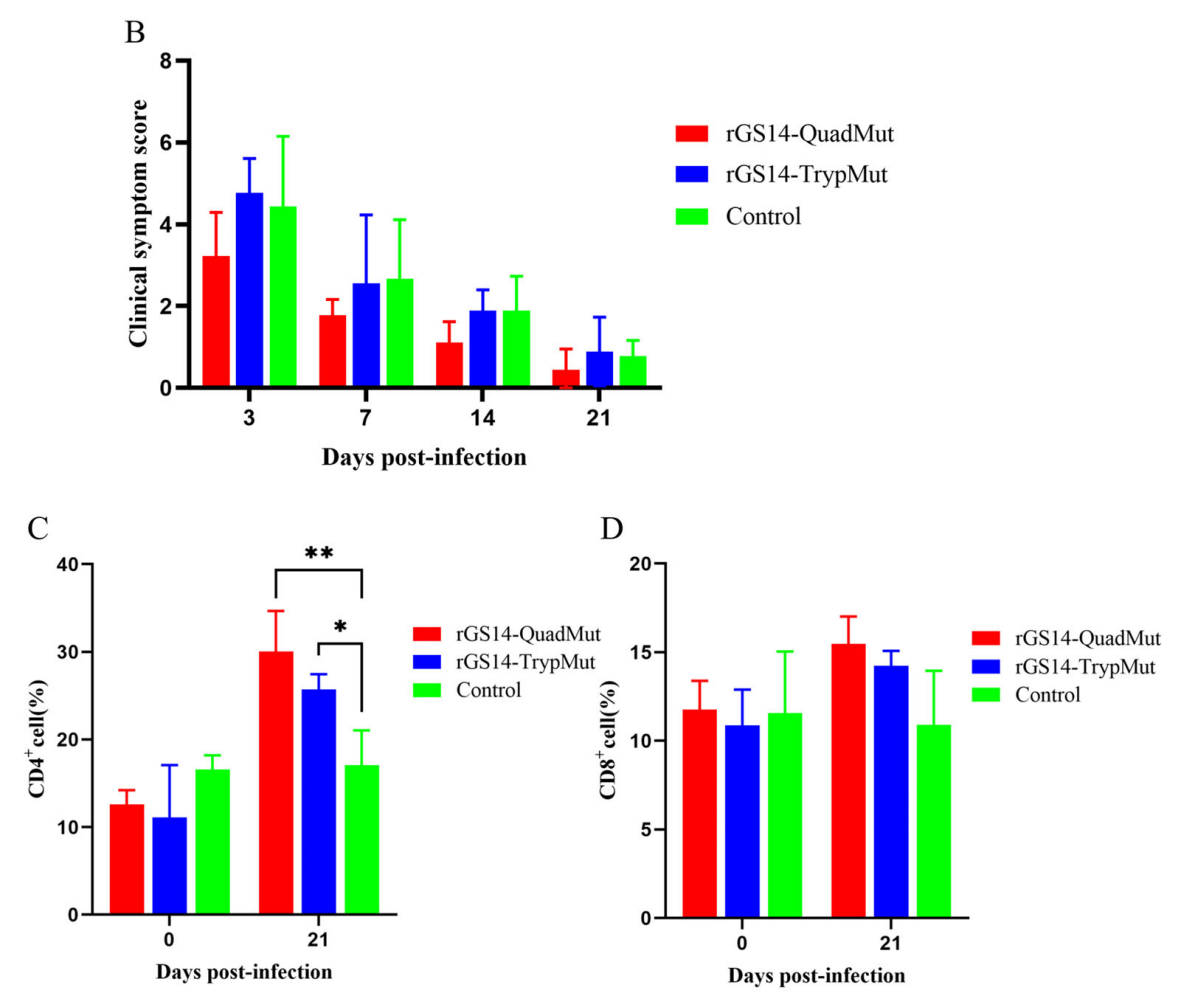
Clinical infection process of rGS14-QuadMut and rGS14-TrypMut. (**A**) Clinical characteristics of lambs immunized with rGS14-QuadMut, rGS14-TrypMut, and DMEM F12 at days 3, 7, 14, and 21 post-inoculation. In the rGS14-QuadMut-immunized group, dark red or brown scabs appeared at the inoculation site on day 3, with no significant inflammation. The wound healed completely by day 7. In contrast, lambs immunized with rGS14-TrypMut exhibited local hemorrhage and swelling at the injection site on day 3, with scabbing beginning on day 7 and the wound healing almost completely by day 14. Each column in the figure represents a separate laboratory animal (*n* = 1); (**B**) bar chart showing clinical symptom scores for lambs immunized with rGS14-QuadMut, rGS14-TrypMut, and DMEM F12; (**C**) flow cytometric analysis of the percentage of CD4^+^ T cells in the blood of lambs before (day 0) and after immunization (day 21); and (**D**) flow cytometric analysis of the percentage of CD8^+^ T cells in the blood of lambs before (day 0) and after immunization (day 21). * *p* < 0.05, ** *p* < 0.01.

To assess the safety of rGS14-QuadMut, rGS14-TrypMut, and DMEM/F12, lambs were immunized and observed for 21 days for clinical changes at the injection site. The results (Figure 3A) show that lambs immunized with the four-gene deletion strain rGS14-QuadMut did not exhibit typical signs of orf lesions. Only dark red or brown scabs were observed at the injection site within 3 days post-inoculation, with no noticeable redness, swelling, or fever. By day 7 post-immunization, the scab had completely fallen off, and the wound healed with normal skin recovery. In contrast, lambs immunized with rGS14-TrypMut displayed significant local inflammation, including hemorrhaging and swelling, at the injection site within 3 days. Scabbing started on day 7, and by day 14, the wound mostly healed. The clinical score data (Figure 3B) show a similar trend for all groups, with no significant differences. The score of the control group was slightly higher than that of the other groups, which may be related to the subjectivity of the scoring method and the error of scratch inoculation operation. These findings indicate that rGS14-QuadMut induces milder local reactions compared to rGS14-TrypMut, suggesting higher safety.

### 3.4. T Cell Subpopulation Analysis

PBMCs were collected from lambs before and 21 days after immunization with rGS14-QuadMut and rGS14-TrypMut. Flow cytometry was used to analyze the changes in CD4^+^ and CD8^+^ T lymphocytes. The results (Figure 3C) show that both rGS14-QuadMut and rGS14-TrypMut-immunized groups exhibited significant changes in the proportion of CD4^+^ T cells compared to the control group. The proportion of CD4^+^ T cells in the rGS14-QuadMut-immunized group was significantly higher than that in the rGS14-TrypMut group. No significant differences in CD8^+^ T cell proportions were observed between the two immunized groups compared to the control. Additionally, the ratio of CD4^+^/CD8^+^ T lymphocytes was higher in the rGS14-QuadMut group than in the rGS14-TrypMut group. These results suggest that both rGS14-QuadMut and rGS14-TrypMut can induce lymphocyte proliferation, with rGS14-QuadMut demonstrating a more prominent effect in promoting CD4^+^ T cell responses.

### 3.5. Cytokine Detection Results

**Figure 4:**
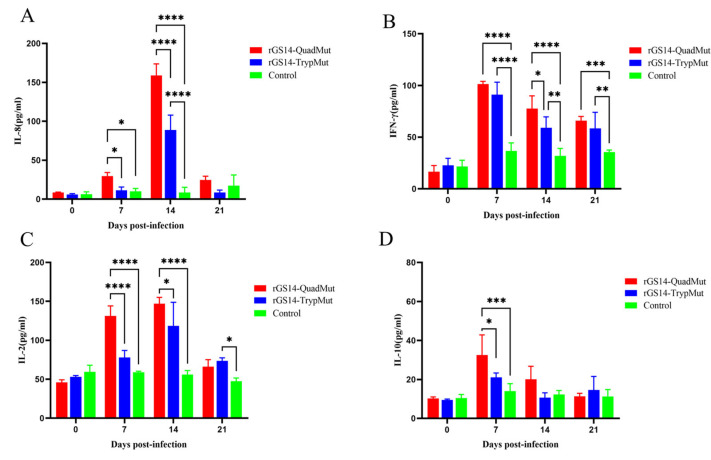
Cytokine secretion levels in lamb serum at 21 Days post-scratch immunization. (**A**) IL-8 levels trend; (**B**) IFN-γ levels trend; (**C**) IL-2 levels trend; and (**D**) IL-10 levels trend. * *p* < 0.05, ** *p* < 0.01, *** *p* < 0.001, and **** *p* < 0.0001.

ELISA kits were used to detect the levels of cytokines of IL-2, IL-10, IL-8, and IFN-γ in the serum of experimental lambs at different time points. The results (Figure 4) show that compared to the DMEM F12 control group, IL-8 levels in rGS14-QuadMut and rGS14-TrypMut-immunized lambs did not increase significantly on day 7, but peaked on day 14, after which they declined. On day 14, IL-8 expression was significantly higher in the rGS14-QuadMut-immunized group compared to the rGS14-TrypMut group (*p* < 0.001). IFN-γ levels increased rapidly after immunization, reaching a peak on day 7, followed by a decline. Compared to the control group, IFN-γ levels were significantly elevated in the rGS14-QuadMut-immunized group (*p* < 0.0001 or *p* < 0.001), and in the rGS14-TrypMut group, IFN-γ levels showed a significant increase on day 7 (*p* < 0.001), with significant differences on days 14 and 21 (*p* < 0.01). IL-2 levels increased slowly after immunization, peaking on day 14 before decreasing. The rGS14-QuadMut-immunized group showed significant increases in IL-2 levels on days 7 and 14, with a highly significant difference compared to the control group (*p* < 0.0001) and a significant difference compared to the rGS14-TrypMut group (*p* < 0.001 or *p* < 0.05). On day 21, IL-2 levels in the rGS14-TrypMut-immunized group increased significantly compared to the control group (*p* < 0.05). No significant difference was observed in the changes in IL-10 cytokine levels between the two immunized groups, but on day 7, IL-10 levels in the rGS14-TrypMut-immunized group were significantly higher than the control group (*p* < 0.05), while IL-10 levels in the rGS14-QuadMut-immunized group showed a highly significant increase (*p* < 0.001).

### 3.6. Results of Challenge Protection Experiment

**Figure 5:**
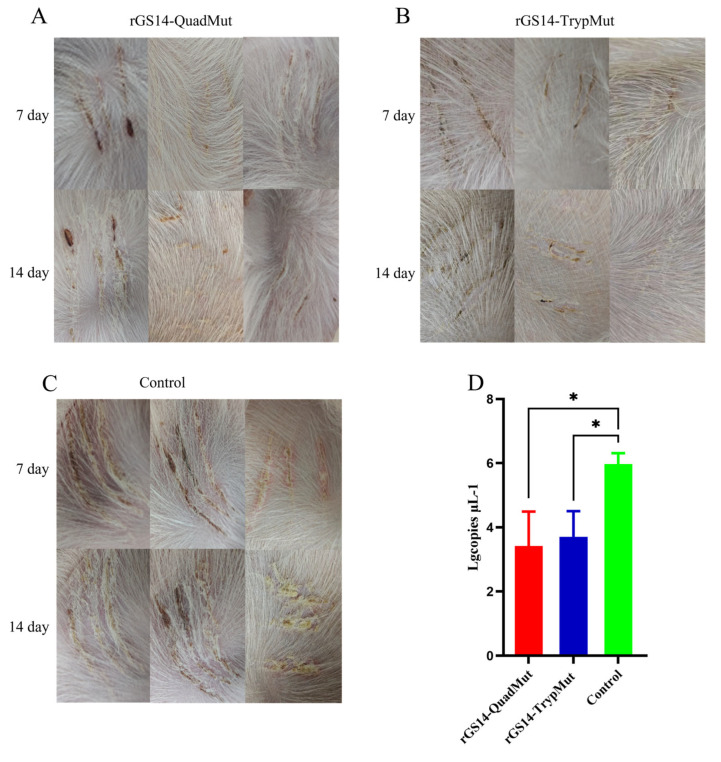

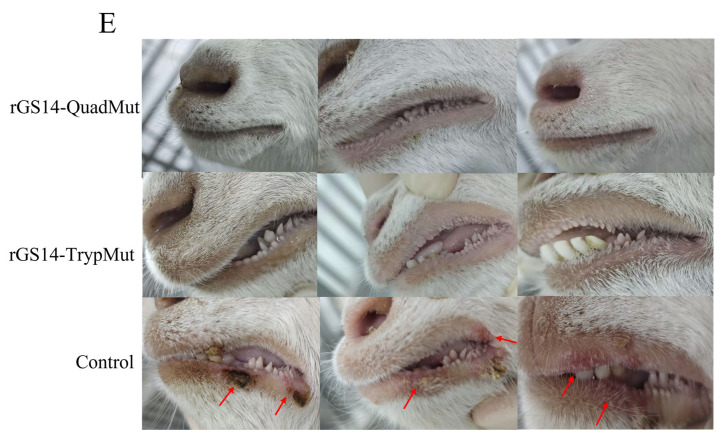
The clinical symptoms of the oral cavity and the scratched inoculation sites of rGS14-QuadMut, rGS14-TrypMut, and control groups after scratching inoculation with ORFV-CQ on the inner thigh of lambs on day 7 and day 14. (**A**) Clinical symptoms in the rGS14-QuadMut group: Only a few lambs showed blood scabs on day 7, with nearly complete recovery by day 14; (**B**) clinical symptoms in the rGS14-TrypMut group: No typical clinical symptoms of orf were observed. Only mild scabbing was seen at the site of the scratch, with full recovery by day 14; and (**C**) clinical symptoms in the control group: pustules and erythema were observed on day 7, followed by scab formation on day 14, showing typical clinical symptoms of orf. (**D**) ORFV viral load map of the crust tissue region at the challenged site on day 14 ∆ the crust tissue region of the rGS14-QuadMut, rGS14-TrypMut, and control groups. (**E**) Clinical symptoms of the rGS14-QuadMut group, rGS14-TrypMut group, and control group in the lips. The arrows indicate the typical clinical symptoms of orf in sheep. * *p* < 0.05.

As shown in Figure 5, the control group that was scratched with the virus showed clear pustules at the inoculation site, which developed into scabs, displaying typical clinical symptoms of orf (CE). However, no typical clinical symptoms of orf were observed at the inoculation sites of lambs immunized with rGS14-QuadMut and rGS14-TrypMut. These results indicate that both rGS14-QuadMut and rGS14-TrypMut provide effective immune protection, preventing the development of typical clinical symptoms following challenge. The results of the qPCR showed that, compared with the control group, the viral content at the challenged sites in the rGS14-QuadMut and rGS14-TrypMut groups was lower, and there were significant differences (*p* < 0.05).

## 4. Discussion

ORFV is a highly infectious pathogen that causes skin diseases in sheep and other ruminants. The virus is classified under the Parapoxvirus genus in the Poxviridae family [24]. ORFV is a double-stranded DNA virus that encodes approximately 130 genes, among which VIL-10 is one of the virulence factors. The protein produced by VIL-10 significantly enhances the virus’s ability to evade the immune system by inhibiting cytokine synthesis, antigen presentation, and T cell activation [17,25]. Previous studies suggested that VIL-10 is a virulence gene, and its deletion typically does not affect the virus’s replication and proliferation capacity [18,26].

In this research, we successfully generated a four-gene deletion strain, rGS14-QuadMut, by knocking out the VIL-10 gene on the backbone of the ORFV three-gene deletion strain using homologous recombination technology. In a previous study, our lab compared the in vitro growth characteristics of rGS14-TrypMut and the parental virulent GS14, and showed that deletions of 121, CBP, and GIF genes did not affect viral proliferation and replication [20]. On this basis, the proliferation and growth characteristics of rGS14-QuadMut, rGS14-TrypMut, and rGS14∆VIL-10 were compared under in vitro. The results demonstrate that all three recombinant viruses exhibited similar levels of viral replication, and the deletion of the VIL-10 gene did not impair viral proliferation, which is consistent with the aforementioned research findings. Furthermore, the genetic stability of rGS14-QuadMut was verified through a continuous passaging experiment of 25 generations in vitro. The results indicate that the recombinant virus maintained stable passaging without genetic variation, demonstrating good genetic stability. These findings provide an experimental foundation for subsequent studies on the safety, immunogenicity, and protective efficacy of rGS14-QuadMut. 

Stability, safety, and the capacity to generate an effective immune response are critical indicators for vaccines [27,28]. In previous studies, our laboratory compared the safety profiles of rGS14-TrypMut and the parental strain GS14, and the results demonstrate that the safety of rGS14-TrypMut was improved [20]. On this basis, in a safety comparison between rGS14-QuadMut and rGS14-TrypMut, using the same dose for scratching inoculation on the inner thigh of lambs, the results show that rGS14-QuadMut lambs were more clinically symptomatic and had a shorter healing time, showing a better safety profile. Deletion of the VIL-10 virulence gene effectively reduces viral virulence, which may be responsible for the reduction in local clinical symptoms.

The T cell immune response is an important indicator of vaccine efficacy, as especially the changes in CD4^+^ T cells and CD8^+^ T cells reflect the responsiveness of the immune system. The results of flow cytometry analysis show that the proportion of CD4^+^ T cells in the rGS14-QuadMut immunization group increased, and the CD4^+^/CD8^+^ ratio increased. This indicates that rGS14-QuadMut can more effectively promote the differentiation of CD4^+^ T cells and enhance the immune response. In cellular immune responses, helper T cells (Th cells) can be divided into Th1 and Th2 subsets, which secrete different cytokines and regulate each other. Th1 cells primarily secrete IFN-γ, IL-2, IL-8, and TNF-α, while Th2 cells secrete IL-4, IL-5, IL-6, IL-10, and IL-13 [29,30]. The results of cytokine assay show that the expression levels of cytokines IL-8, IFN-γ, and IL-2 were significantly increased in the rGS14-QuadMut immune group, indicating that the immune group could induce a strong immune response. Among them, the high level of IL-10 expression is related to the properties of ORFV itself. The virus invades the host and continues to multiply, and the host cells continue to secrete IL-10, inhibiting the cellular immune response [31]. The VIL-10 gene has been shown to inhibit the production of TNF-α, IFN-γ, and IL-8 cytokines, while the ORFV 121 gene encodes a novel NF-κB inhibitor that mediates a severe skin inflammatory response, leading to increased secretion of IFN-γ and IL-2 [32,33]. Consistent with the experimental results, further analysis of the experimental results showed that on the 14th day after immunization, the expression level of IL-8 in the rGS14-QuadMut immunization group was significantly higher than that in the rGS14-TrypMut immunization group, and the high level of IL-8 expression was associated with the deletion of the VIL-10 gene, and the expression levels of IL-2 and IFN-γ cytokines first increased and then decreased, reaching a peak on day 7, which further proved that rGS14-QuadMut effectively activates the Th1 immune response.

In the challenge protection experiment, both rGS14-QuadMut and rGS14-TrypMut-immunized groups did not show typical clinical symptoms of orf at the scratch inoculation site and lips, while the control group developed pustules and other typical pathological features of orf at the scratch inoculation site and lips, indicating that both rGS14-QuadMut and rGS14-TrypMut effectively induced immune protection. However, in previous laboratory studies, GS14 failed to produce the desired immune protection effect after immunizing lambs, which also showed that rGS14-QuadMut and rGS14-TrypMut had significant advantages in immune protection compared with the parental poison GS14. The results of the detection of ORFV load in the tissues of the scratch inoculation site show that the rGS14-QuadMut and rGS14-TrypMut immunization groups were significantly lower than those of the control group. In conclusion, rGS14-QuadMut exhibits a higher safety profile and elicits a stronger immune response while providing equally effective immune protection compared to rGS14-TrypMut. Thus, rGS14-QuadMut holds potential as a candidate strain for a live attenuated orf vaccine. 

## 5. Conclusions

This study successfully constructed the orf virus quadruple gene-deletion strain rGS14-QuadMut. The in vitro growth characteristics of rGS14-QuadMut were validated, demonstrating that the deletion of the VIL-10 gene has minimal impact on its proliferation and replication, and the strain exhibits good stability. The safety of rGS14-QuadMut is superior to that of rGS14-TrypMut, primarily inducing a Th1-type cellular immune response while providing equally effective immune protection.

## Figures and Tables

**Table 1:** Primer sequences.

Primer Name	Sequence Information(5’-3’)
VIL-10-F	AACAACAAAATTCTGGTG
VIL-10-R	TGTAGTTGATGAATATGTCG
VIL-10-hm1-F	CGTGGACCCCGCTGTGCTGAAGAC
VIL-10-hm1-R	AACAGGTGCGGTGAACTCGAAGACGC
VIL-10-hm1-egfp-R	GGCACGTAGAAGACCAGGAAACAGGT GCGGTGAACTCGAAGACGC
VIL-10-hm2-egfp-F	TGTCCAAACTCATCAATGTATCTTAATTC ATCAACTACATAGAATCAT
VIL-10-hm2- R	CGACAGCCAGCAGCGGCCCCACCAG
egfp-VIL-10-hm1-F	GCGTCTTCGAGTTCACCGCACCTGTTTC CTGGTCTTCTACGTGCC
egfp-VIL-10-hm2-R	ATGATTCTATGTAGTTGATGAATTAAGA TACATTGATGAGTTTGGACA
VIL-10-hm2-F	ATTCATCAACTACATAGAATCAT
VIL-10-hm1-hm2-F	CACAGACCTGCTCGACAACA
VIL-10-hm1-hm2-R	GTATGATTCTATGTAGTTGATGAAGCATGTGCGGTGAACTCGAA
VIL-10-hm2-hm1-F	GTCTTCGAGTTCACCGCACATGCTTCATCAACTACATAGAATCA
VIL-10-hm2-hm1-R	CAGCCGTGGCGGATGA

## Data Availability

Informed consent was obtained from all subjects involved in the study.

## Supplementary Materials

The following supporting information can be downloaded at: https://www.mdpi.com/article/10.3390/v17060760/s1, Figure S1: Purification process of rGS14-QuadMut-GFP; Figure S2: Verification of VIL-10 gene knockout in rGS14-QuadMut-GFP. VIL-10 PCR amplification products; Figure S3: Purification process of rGS14-QuadMut; Figure S4: Verification of VIL-10 gene knockout in rGS14-QuadMut. VIL-10 gene PCR amplification products.
