# Supplementary material for: Construction and Biological Characteristics of a Quadruple Gene-Deleted Strain of Orf Virus as a Vaccine Candidate
Source: Viruses. 2025 May 27;17(6):760. doi: 10.3390/v17060760 (PMC12197802; doi:10.3390/v17060760)
Supplement: Supplementary file 1 [file viruses-17-00760-s001.zip › viruses-3542310-supplementary.pdf]

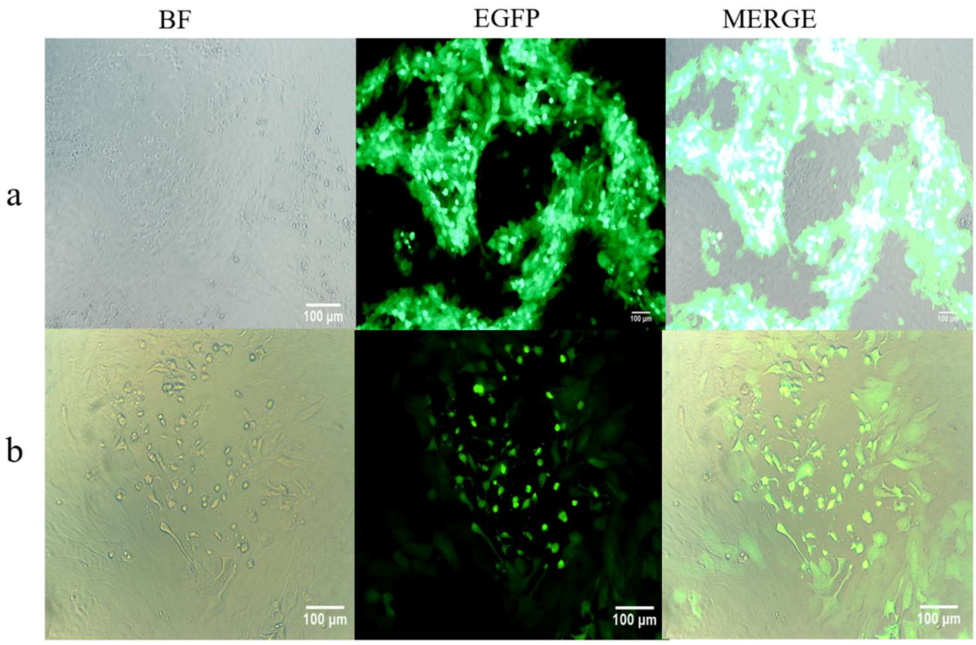

Figure S1: Purification process of rGS14-QuadMut-GFP, (a) second round, (b) three round;

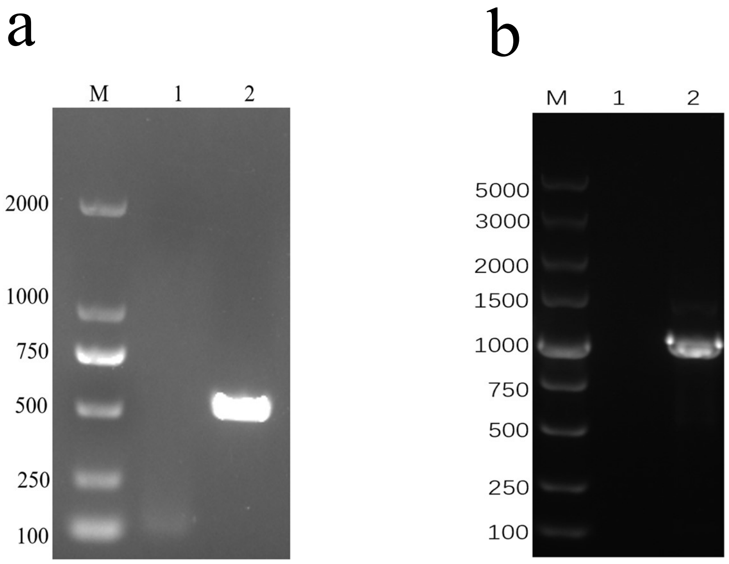

Figure S2 (a) Verification of VIL-10 gene knockout in rGS14-QuadMut-GFP. VIL-10 PCR amplification products. M: DL 2000 bp DNA ladder; 1: PCR amplification product; 2: Positive control;

(b) Verification of GFP gene knockout in rGS14-QuadMut-GFP. GFP PCR amplification products. M: DL 5000 bp DNA ladder; 1: Negative control; 2: PCR amplification product;

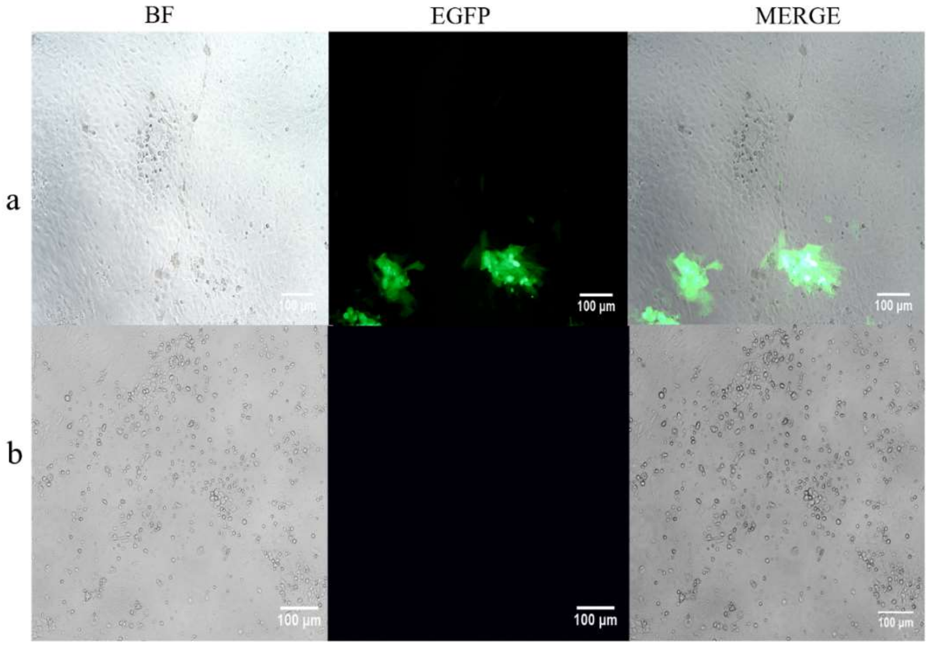

Figure S3: Purification process of rGS14-QuadMut, (a) second round, (b) fifth round;

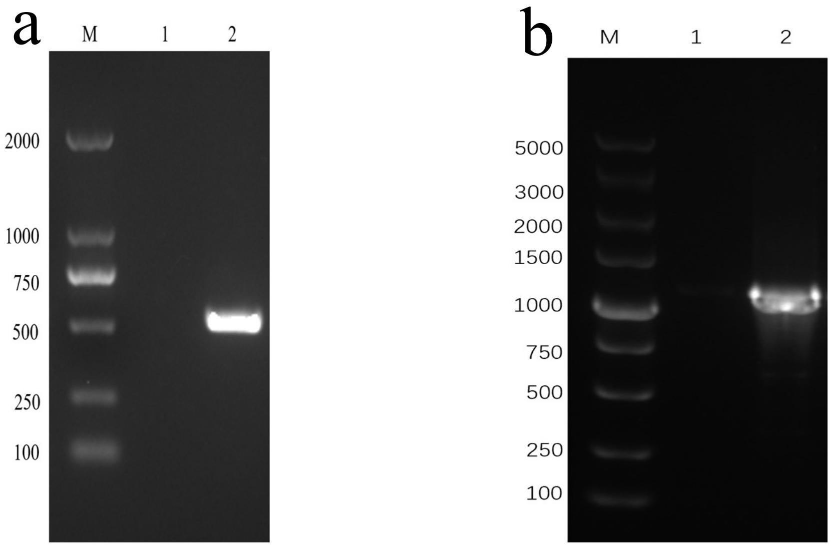

Figure S4 (a) Verification of VIL-10 gene knockout in rGS14-QuadMut. VIL-10 gene PCR amplification products. M: DL 2000 bp DNA ladder; 1: PCR amplification product; 2: Positive control;

(b) Verification of GFP gene knockout in rGS14-QuadMut. GFP gene PCR amplification products. M: DL 5000 bp DNA ladder; 1: PCR amplification product; 2: Positive control;
